# Supplementary material for: Structural insights into Siglec-15 reveal glycosylation dependency for its interaction with T cells through integrin CD11b
Source: Nat Commun. 2023 Jun 13;14:3496. doi: 10.1038/s41467-023-39119-8 (PMC10264397; doi:10.1038/s41467-023-39119-8)
Supplement: Supplementary file 1 — Supplementary Information [file 41467_2023_39119_MOESM1_ESM.pdf]

SUPPLEMENTARY INFORMATION

Structural insights into Siglec-15 reveal glycosylation dependency for its interaction with T cells through integrin CD11b

Maria Pia Lenza[1,11], Leire Egia-Mendikute[2,11], Asier Antoñana-Vildosola[2,11], Cátia O. Soares[3,4], Helena Coelho[3,4], Francisco Corzana[5], Alexandre Bosch[2], Prodhi Manisha[2,] Jon Imanol Quintana[1], Iker Oyenarte[1], Luca Unione[1,6], María Jesús Moure[1], Mikel Azkargorta[7], Unai Atxabal[1], Klaudia Sobczak[1], Felix Elortza[7], James D. Sutherland[8], Rosa Barrio[8], Filipa Marcelo[3,4], Jesús Jiménez-Barbero[1,6,9,10,*], Asis Palazon[2,6,*], June Ereño-Orbea[1,6,12,*]

[1]Chemical Glycobiology lab, Center for Cooperative Research in Biosciences (CIC bioGUNE), Basque Research and Technology Alliance (BRTA), Bizkaia Technology Park, Building 800, 48160 Derio, Bizkaia, Spain.

[2]Cancer Immunology and Immunotherapy Lab, Center for Cooperative Research in Biosciences (CIC bioGUNE), Basque Research and Technology Alliance (BRTA), Bizkaia Technology Park, Building 801A, 48160 Derio, Bizkaia, Spain.

[3] Associate Laboratory i4HB - Institute for Health and Bioeconomy, NOVA School of Science and Technology, Caparica campus 2829-516 Caparica, Portugal.

[4] UCIBIO, Department of Chemistry, NOVA School of Science and Technology, Caparica campus 2829-516 Caparica, Portugal

[5] Department of Chemistry, University of La Rioja, The Center for Research in Chemical Synthesis, Madre de Dios 53, E-26006 Logroño, Spain.

[6] Ikerbasque, Basque Foundation for Science, Bilbao, Spain.

[7]Proteomics Platform, CIC bioGUNE, CIBERehd, Basque Research and Technology Alliance (BRTA), Bizkaia Technology Park, Building 800, 48160 Derio, Spain.

[8]Ubiquitin-likes and Development Lab, Center for Cooperative Research in Biosciences (CIC bioGUNE), Basque Research and Technology Alliance (BRTA), Bizkaia Technology Park, Building 801A, 48160 Derio, Bizkaia, Spain.

[9]Department of Organic & Inorganic Chemistry, Faculty of Science and Technology, University of the Basque Country, EHU-UPV, 48940 Leioa, Bizkaia, Spain.

[10]Centro de Investigacion Biomedica En Red de Enfermedades Respiratorias, 28029 Madrid, Spain.

[11]These authors contributed equally.

[12]Lead contact.

*Correspondence: Jesús Jiménez-Barbero (jjbarbero@cicbiogune.es), Asis Palazon (apalazon@cicbiogune.es), June Ereño-Orbea (jereno@cicbiogune.es)

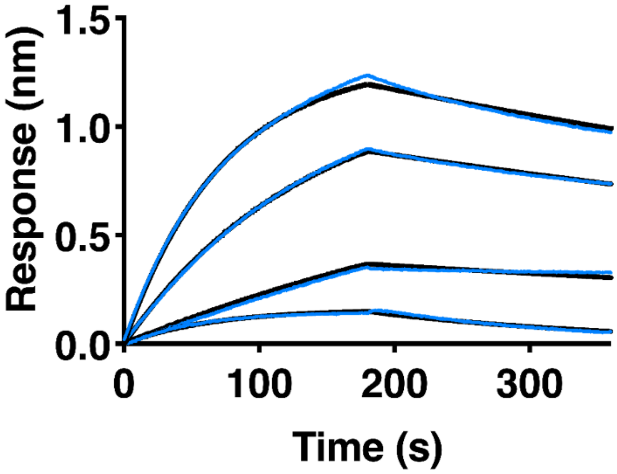

K_D (nM)	K_{on} (10^5 1/Ms)	K_{off} (10^{-3} 1/Ms)
4.68 ± 0.29	2.72 ± 0.11	0.001 ± 0.0001

Supplementary Fig. 1. 5G12 Fab binding to recombinant Siglec-15. Representative sensograms for 5G12 Fab binding to Siglec-15-Fc (blue) and the 1:1 models that provide the best fits (black), calculated by BLI. The Table gathers the mean values of the kinetic parameters with the standard errors (SEM, representative of three independent measurements).

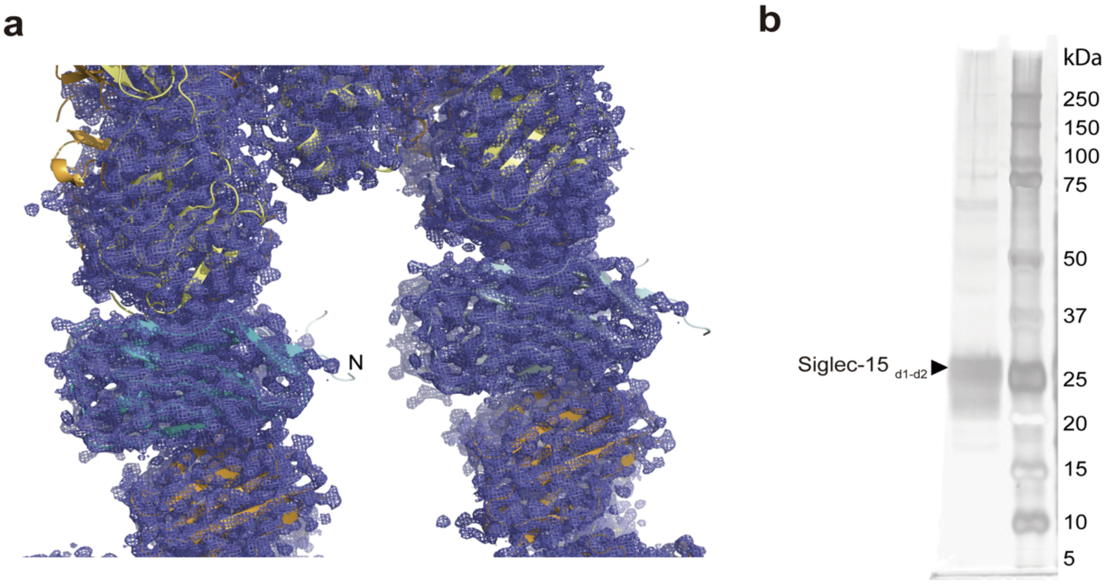

Supplementary Fig. 2. Electron density map and SDS-PAGE gel for the Siglec-15$_{d1d2}$ and 5G12 Fab complex crystal structure. a View of the composite omit map contoured at 1σ of the area, where the C-terminal end of Siglec-15 V-set domain is located. **b** Diffracting crystals were run on SDS-PAGE gel and marked with silver staining. The molecular weight markers (in kDa) are indicated on the right.

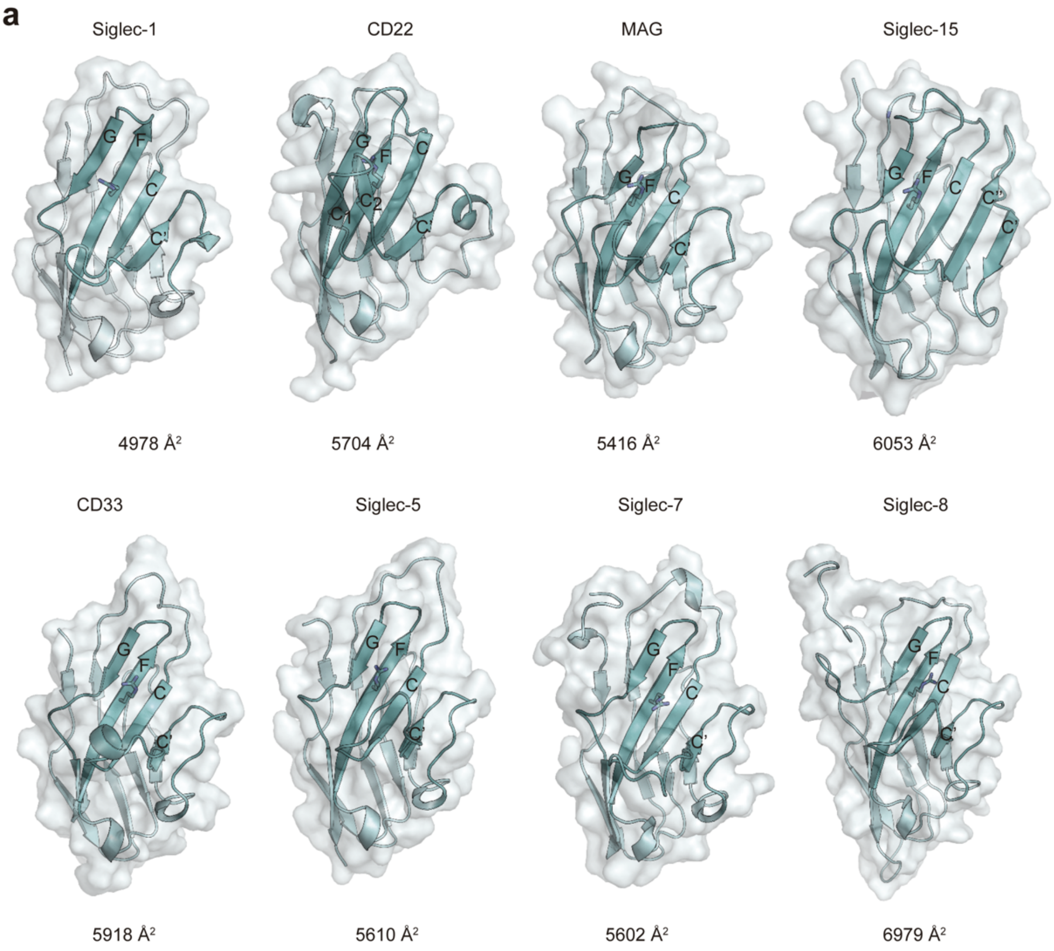

b

	Siglec-1	CD22	MAG	CD33	Siglec-5	Siglec-7	Siglec-8
**Siglec-15**	0.723	4.476	1.151	1.766	1.375	1.498	1.324

Supplementary Fig. 3. Structural comparison of the V-set domains of Siglecs. a Cartoon and surface representation of the CFG β-sheet side view of the V-set domains from Siglec-1 (PDB ID: 1QFP), CD22 (PDB ID: 5VKJ), MAG (PDB ID: 5LFR), CD33 (PDB ID: 5IHB), Siglec-5 (PDB ID: 2ZG2), Siglec-7 (PDB ID: 1O7S) and Siglec-8 (PDB ID: 2N7A). The surface area of the CFG β-sheet was calculated with Pymol[1] and the values are depicted under each structure. **b** The Cα r.m.s.d. values of the three-dimensional structure of Siglec-15 V-set domain fold when compared to other Siglecs, as calculated with Pymol[1].

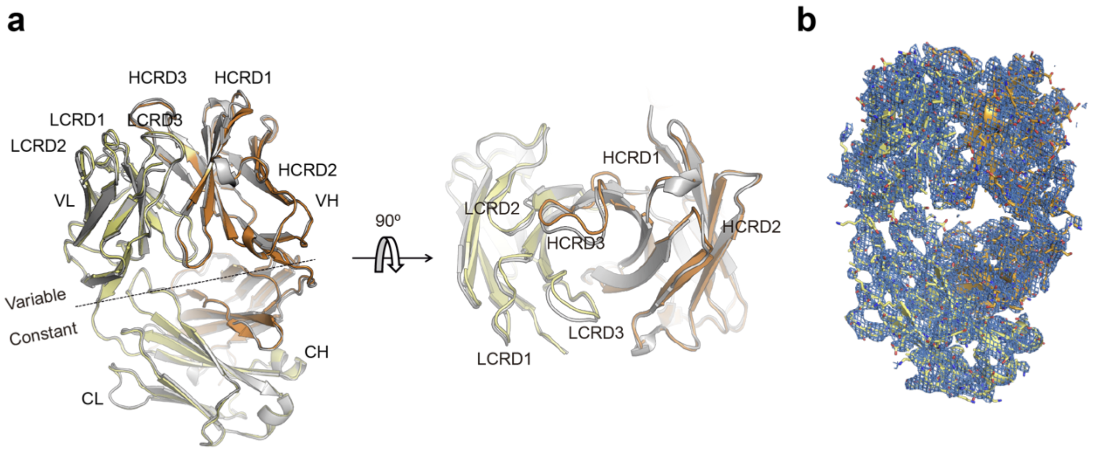

Supplementary Fig. 4. 5G12 Fab crystal structures. a Superposition of the 5G12 Fab crystal structures in the unliganded (in grey) and bound (in yellow the LC and in orange the HC) forms. Cartoon representation of the Fab portion of 5G12 Ab composed of the variable light (VL) and heavy (VH) chain domains; and the constant light (CL) and heavy (CH) chain domains (left). The heavy and light chain variable regions are shown, together with the complementarity-determining regions (CRDs) (right). **b** View of the 2|Fo|-|Fc| electron density map at 1σ contour level and carve radius 1.8 Å of one of the molecules of 5G12 Fab present in the asymmetric unit.

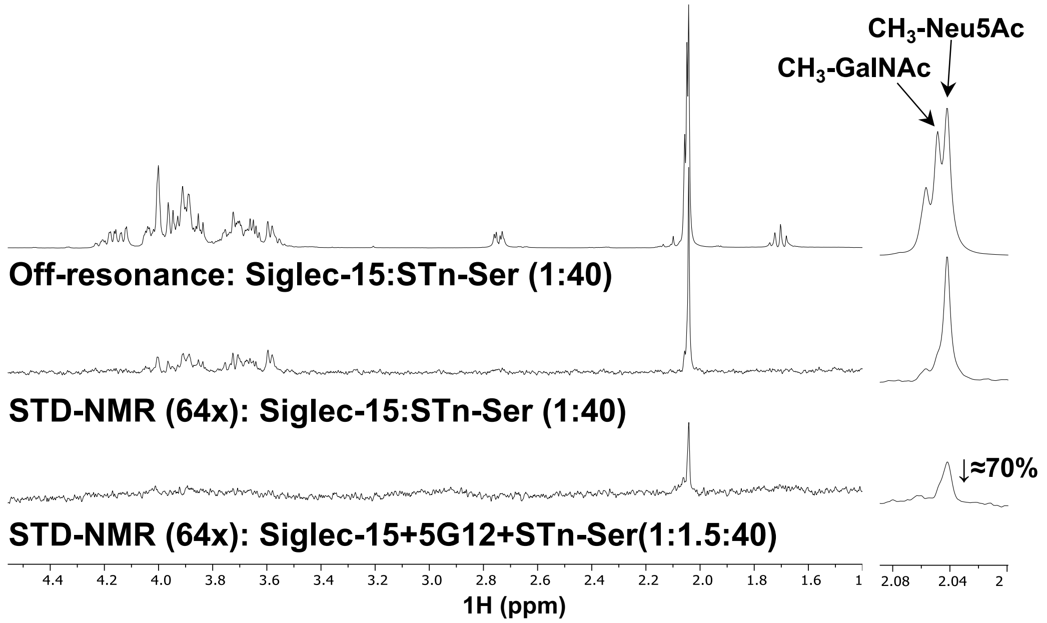

Supplementary Fig. 5. Anti-Siglec-15 blocking mAb 5G12 competes for the sialic acid binding site of Siglec-15. Reference off-resonance spectrum (top) and STD-NMR spectrum (middle) of the Siglec-15 + STn-Ser mixture (molar ratio 1:40). STD-NMR spectrum (bottom) of the Siglec-15 + 5G12 + STn-Ser mixture (molar ratio 1:1.5:40). All spectra were acquired in a 600 MHz spectrometer at 283 K. The STD-NMR spectra were obtained after subtracting the protein controls spectra.

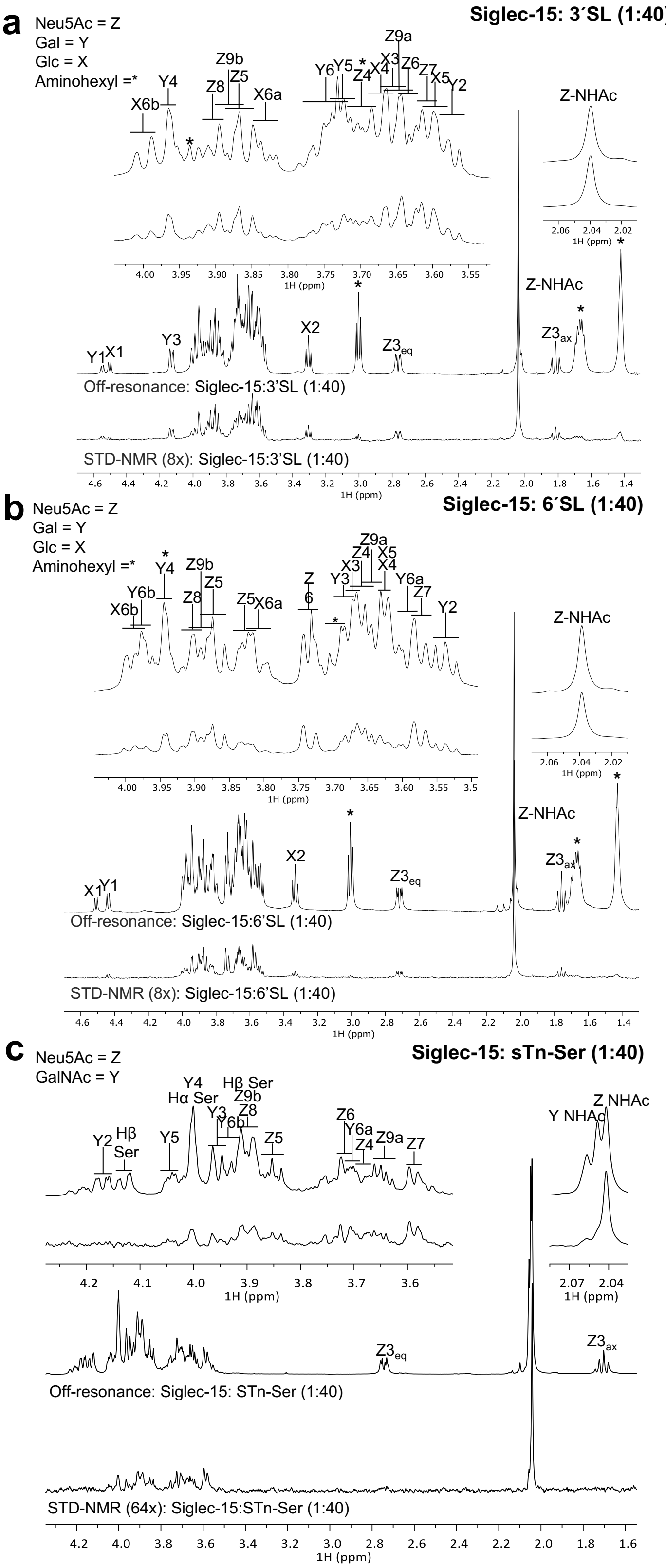

Supplementary Fig. 6. STD-NMR spectra of 3´SL, 6´SL and STn-Ser in presence of Siglec-15. **a** STD NMR experiment of 3´SL in presence of Siglec-15 with 40:1 molar ratio (800 µM 3'SL: 20 µM Siglec-15) acquired at 600 MHz spectrometer and at 283 K. **b** STD experiment of 6´SL in presence of Siglec-15 with 40:1 molar ratio (800 µM

6’SL: 20 μM Siglec-15) acquired at 600 MHz spectrometer and at 283 K. The off-resonance spectra in a and b contains the assignment of the ligand protons (Neu5Ac = Z, Gal = Y, Glc = X, aminohexyl = *). **c** STD experiments of STn-Ser/Siglec-15 with 40:/1 molar ratio (800 μM STn-Ser/20 μM Siglec-15) acquired at 600 MHz spectrometer and at 283 K. The off-resonance spectrum contains the assignment of the ligand protons (Neu5Ac = Z, GalNAc = Y, Serine = Ser). The STD-NMR spectra in all cases had the protein control spectrum subtracted.

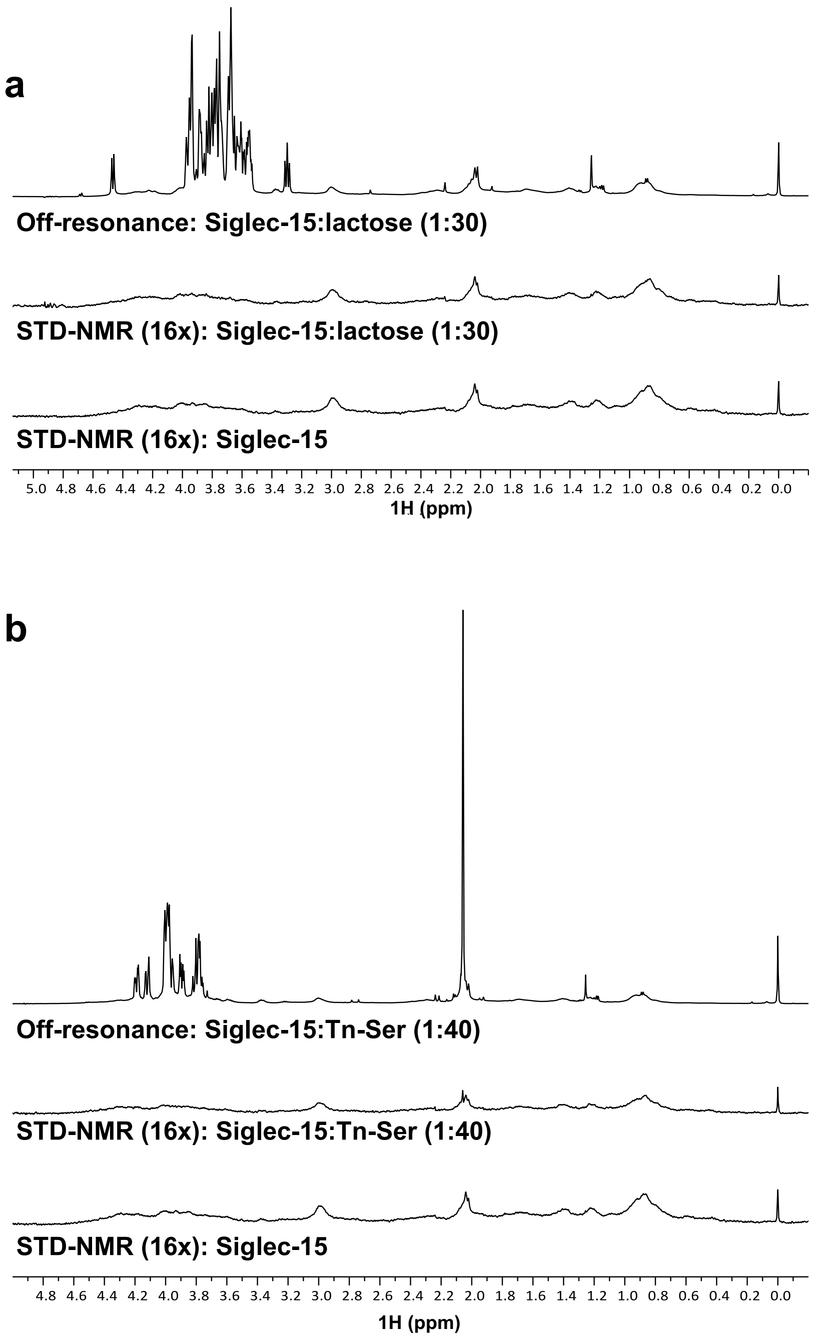

Supplementary Fig. 7. Neu5Ac is required for binding to Siglec-15. a Off-resonance (top) and STD-NMR spectra (middle) of lactose in presence of Siglec-15 (at 1:30 molar ratio; Siglec-15 (30 µM):lactose (900 µM)). **b** Off-resonance (top) and STD-NMR spectra (middle) of Tn-Ser in presence of Siglec-15 (at 1:40 molar ratio; Siglec-15 (20 µM):Tn-Ser (800 µM)). STD-NMR spectrum (bottom) of Siglec-15 (30 µM) in absence of any ligand. All spectra were acquired at a 600 MHz spectrometer and at 283 K. The STD of Siglec-15 in absence of any ligand is identical than those STD obtained for lactose or Tn-Ser in presence of Siglec-15.

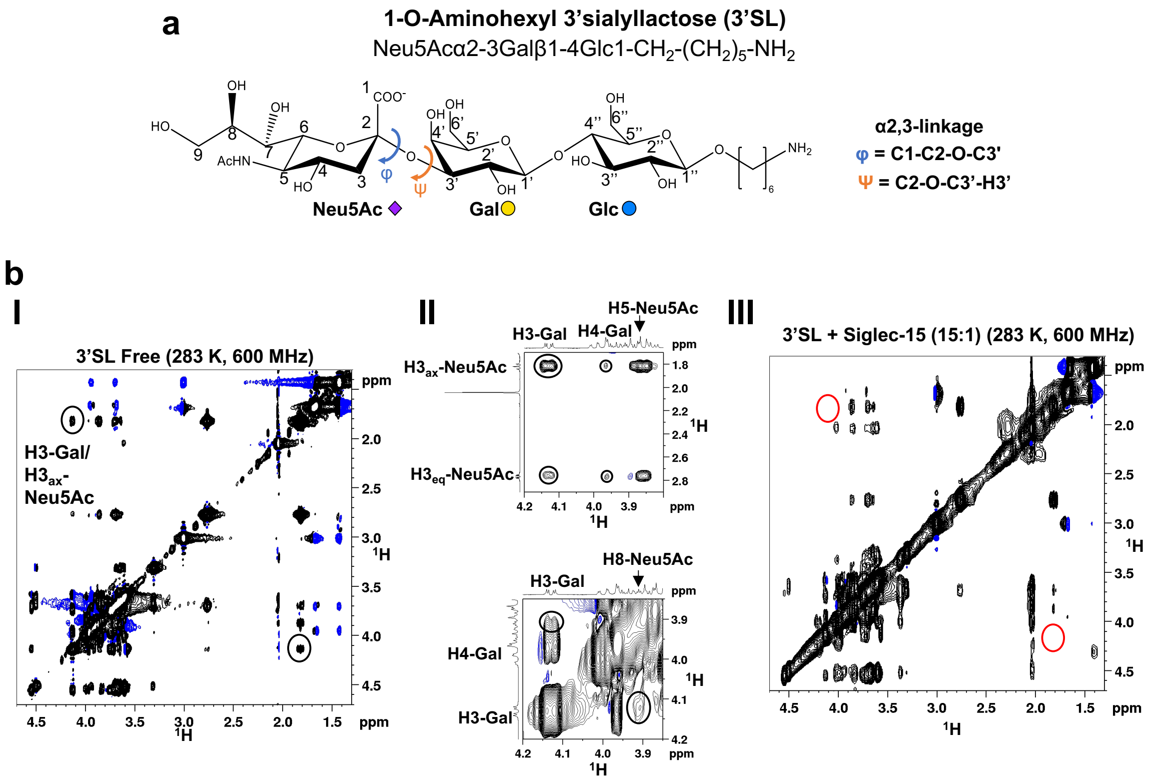

Supplementary Fig. 8. NOESY spectra of 3'SL in absence and presence of Siglec-15. a The structure of 3'SL and definition of the torsion angles around α(2,3)-Neu5Ac linkage. **b** NOESY spectrum of 3'SL in the free state (450 μM, mixing time 400 ms, 600 MHz, 283 K) in full view (I) and relevant zoom in view (II). The co-existence of the H3-Gal,H3ax-Neu5Ac NOE and H3-Gal,H8-Neu5Ac indicates the presence of t and –g conformer, respectively. Tr-NOESY spectra for the conformational analysis of 3'SL in the bound state to Siglec-15 (III) (molar ratio of 1:15 (30μM Siglec-15 : 450 μM 3'SL), mixing time of 150 ms, at 600 MHz and 283 K). The absence of H3-Gal,H3ax-Neu5Ac NOE in the bound state exclude the existence of this conformer in the bound state. Thus, in bound state –g conformer is the major conformer.

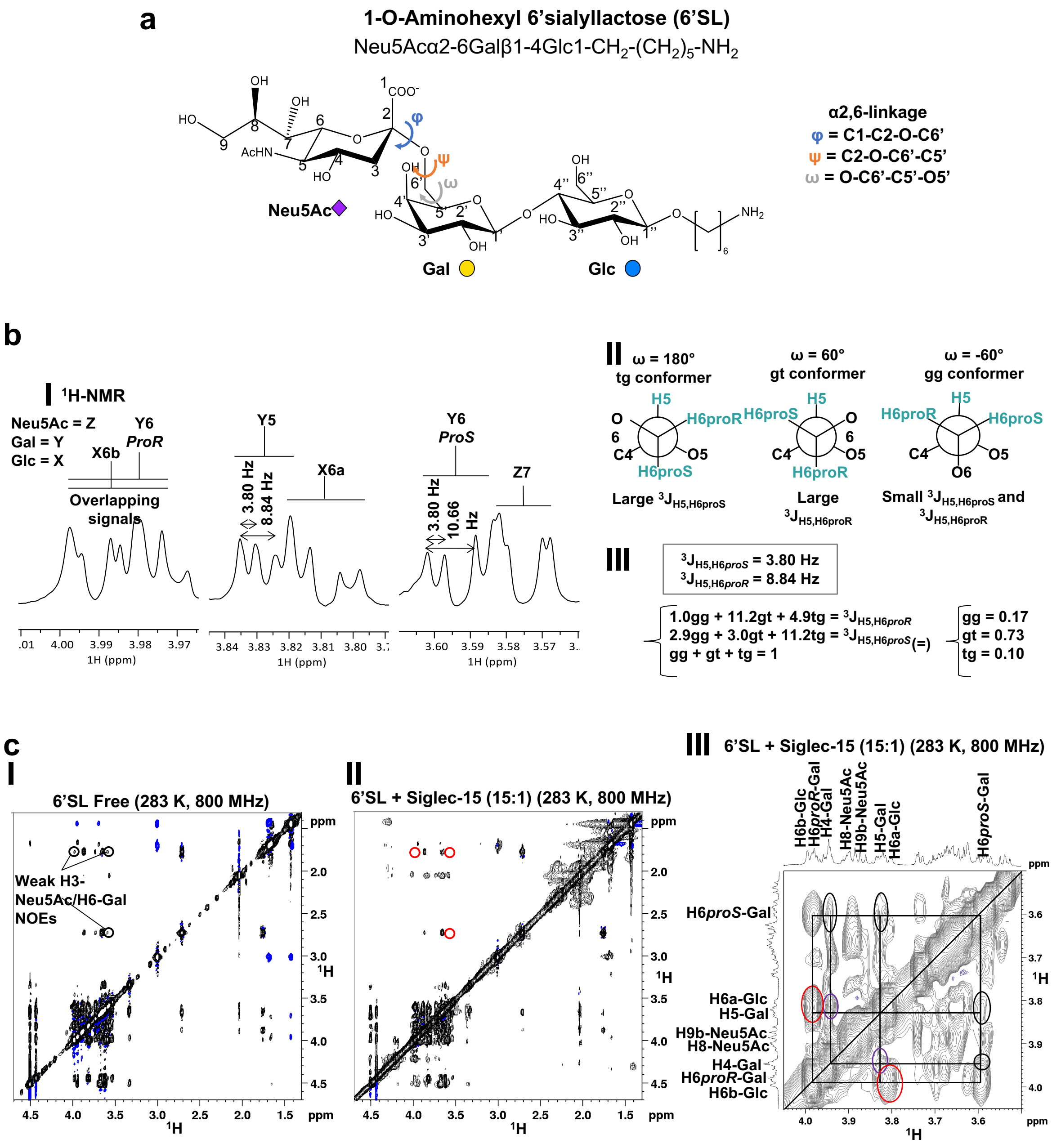

Supplementary Fig. 9. NOESY spectra of 6'SL in absence and presence of Siglec-15. a The structure of 6'SL and definition of the torsion angles around α(2,6)-Neu5Ac linkage. **b** Conformational analysis of 6'SL in the free state using $^3J_{H5,H6\ Gal}$ coupling constants. (I) Regions of the 1H-NMR spectrum of 6'SL at 900 μM in 10 mM phosphate buffer, 200 mM NaCl, D_2O at pH 7.4, acquired at 800 MHz and 283 K, with lb = 0.3 Hz, with coupling constants measurements. (II) Newman projections for the three rotamers around ω torsion angle around the C5-C6 bond of Gal. (III) System of equations to determine the time-averaged distributions of ω conformers of

Neu5Acα(2,6)-Gal sialosides [2] (Ohrui et al, 1991) In solution the major conformer of 6'SL around ω is the gt conformer (60º). **c** (I) NOESY spectrum of 6'SL in the free state (900 μM, mixing time 400 ms, 800 MHz, 283 K). The weak NOEs between H3-Neu5Ac and H6(s)Gal protons suggest that the major conformer around φ is the –g conformer (-60º). (II) Tr-NOESY spectrum with a molar ratio of 1:15 (30μM Siglec-15 : 450 μM 6'SL), mixing time of 150 ms, at 800 MHz and 283 K. The absence of NOEs between H3-Neu5Ac and H6(s)Gal protons in the bound state indicates that the major conformer around φ is the –g conformer (-60º). (III) Zoom in view of tr-NOESY region, where black lines are guides to find the NOE cross-peaks concerning H4 and H5 interactions with H6*proS* and H6*proR* from Gal (circled in black). Red circles indicate overlapping strong NOEs cross-peaks (H6a/H6b Glc). Purple circles indicate references for strong NOE cross-peaks (H4/H5 Gal). This analysis exclude the gg conformer in the case of 6'SL, but did not allow to discriminate the relative population between gt and tg conformers in the bound state.

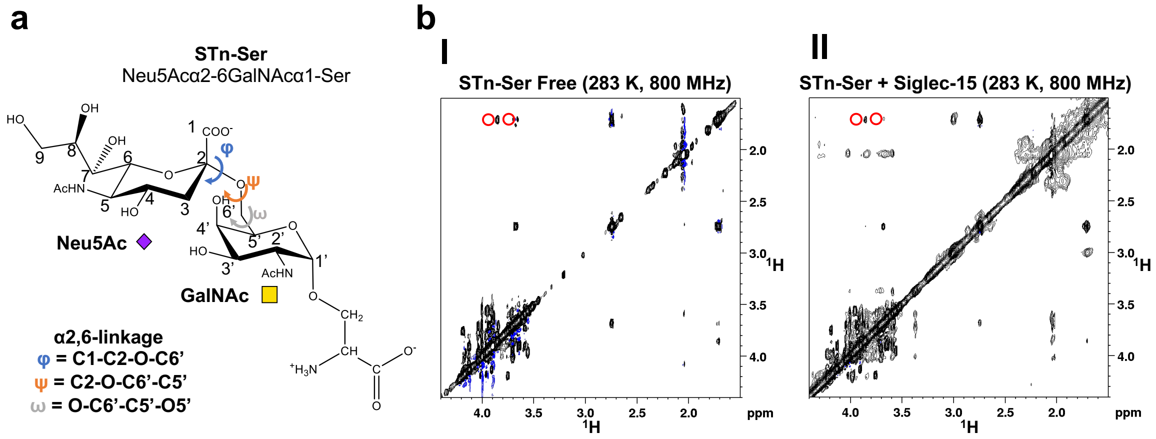

Supplementary Fig. 10. NOESY spectra of STn-Ser in absence and presence of Siglec-15. **a** The structure of STn-Ser and definition of the torsion angles around α(2,6)-Neu5Ac linkage. **b** NOESY spectra of STn-Ser in absence and presence of Siglec-15. (I) NOESY spectrum of STn-Ser in the free state (450 μM, mixing time 400 ms, 800 MHz, 283 K). (II) Tr-NOESY spectrum of STn-Ser in the presence of Siglec-15, with 15:1 molar ratio (450 μM STn-Ser; 30 μM Siglec-15), mixing time of 150 ms, at 800 MHz and 283 K. The red circles indicate the absence of NOEs between H3ax-Neu5Ac and H6s-GalNAc, which indicate the predominance of the -g conformer around the φ dihedral angle (-60°).

a

Siglec-15 + STn-Ser	
Center x	13.02
Center y	-3.73
Center z	29.52
Size x	34
Size y	52
Size z	16
Exhaustiveness	200
Num. modes	20
Energy range	4

b

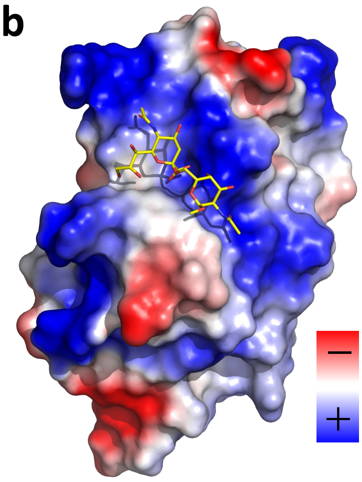

c

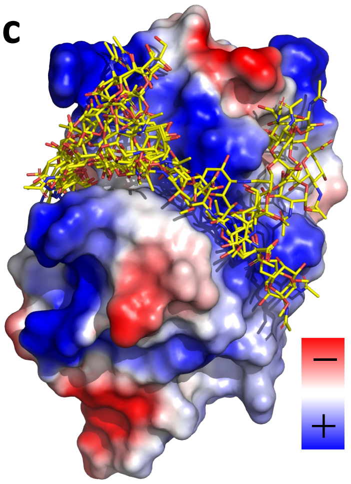

d

Pose	Affinity (kcal/mol)	RMSD (Å, lower bound)	RMSD (Å, upper bound)
**1**	-6.1	0.000	0.000
**2**	-5.9	7.530	13.204
**3**	-5.8	8.518	11.787
**4**	-5.8	4.245	8.132
**5**	-5.8	4.769	9.935
**6**	-5.7	5.884	11.087
**7**	-5.7	3.484	10.207
**8**	-5.7	9.143	12.657
**9**	-5.7	2.942	5.738
**10**	-5.6	4.175	10.232
**11**	-5.6	4.218	8.047
**12**	-5.6	3.791	8.283
**13**	-5.6	4.524	8.582
**14**	-5.6	3.833	7.883
**15**	-5.6	2.411	9.242
**16**	-5.5	9.944	11.689
**17**	-5.5	3.776	9.476
**18**	-5.5	12.566	16.775
**19**	-5.4	12.039	14.791
**20**	-5.4	7.538	13.642

Supplementary Fig. 11. Docking calculations for Siglec-15 crystal structure and STn ligand. a Input file used in AutoDock Vina 1.2.0. **b** Proposed binding pose (pose #1) of α(2,6)Neu5Ac-GalNAc-OMe (stick) in complex with Siglec-15 calculated with AutoDock Vina 1.2.0. The electrostatic potential surface of the protein, estimated with PyMOL 2.5.3 [1] is also shown. **c** 20 best scored poses, in terms of binding energy, of Neu5Acα(2-6)-GalNAc-OMe in complex with Siglec-15 calculated by AutoDock Vina 1.2.0. **d** Poses, affinity and root-mean-square deviation (RMSD) derived from the docking calculations.

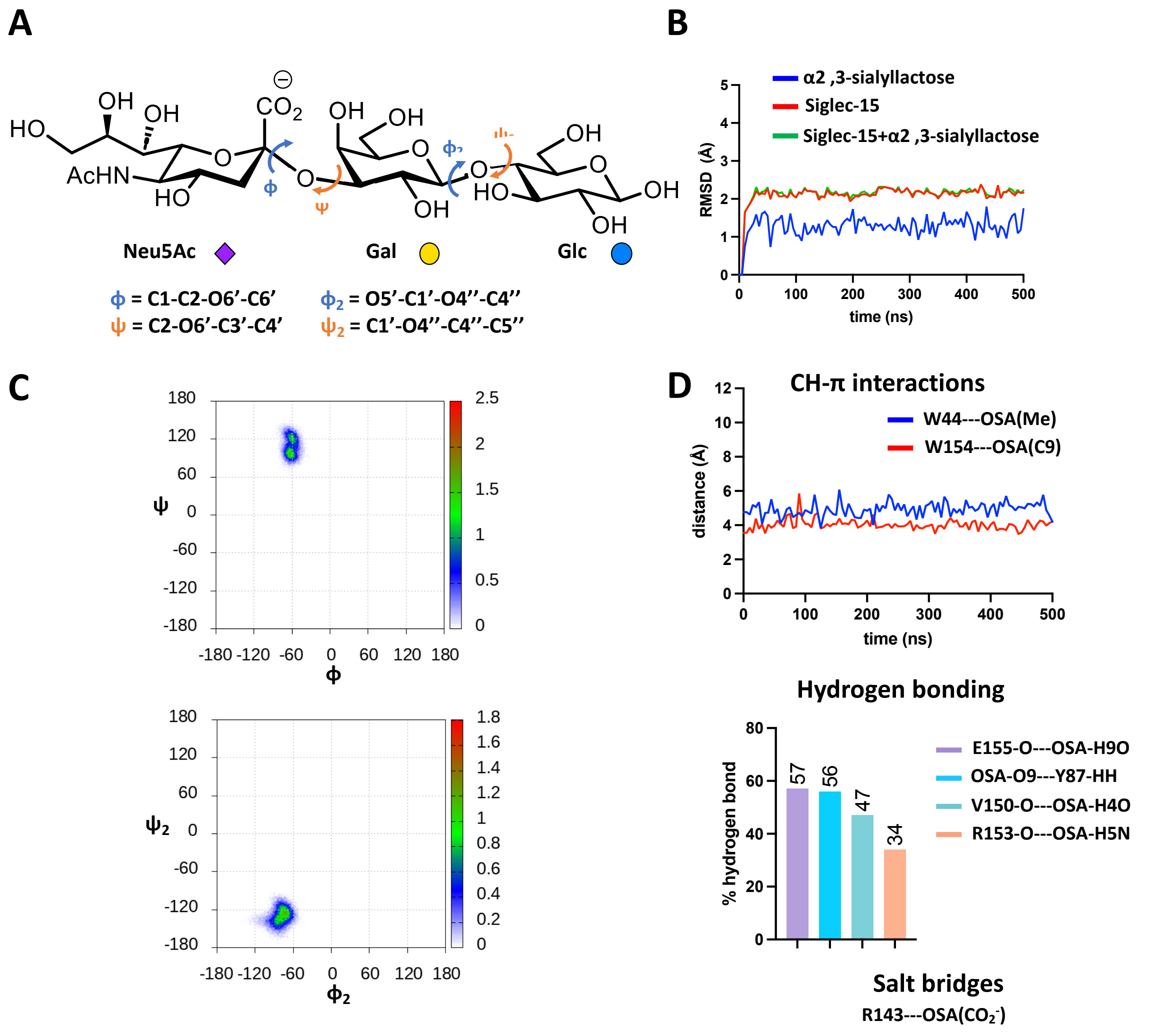

Supplementary Fig. 12. Molecular dynamics (MD) simulations of Siglec-15 in complex with 3'SL. a Structure of 3'SL along with the definition of the torsion angles Φ/ψ and Φ $_2$/ψ $_2$. **b** RMSD (root-mean-square deviation) plots for 3'SL, Siglec-15 and complex Siglec-15/3'SL along 500 ns MD simulations. **c** Φ/ψ distribution around α(2,3)Neu5Ac-GalNAc linkage and Φ$_2$/ψ$_2$ distribution around Galβ(1-4)Glc linkage during 500 ns MD simulations. **d** Molecular interactions (CH-π, hydrogen bonding, salt bridges) along 500 ns MD simulations. The center of the aromatic ring and the center of the sugar were considered in these calculations.

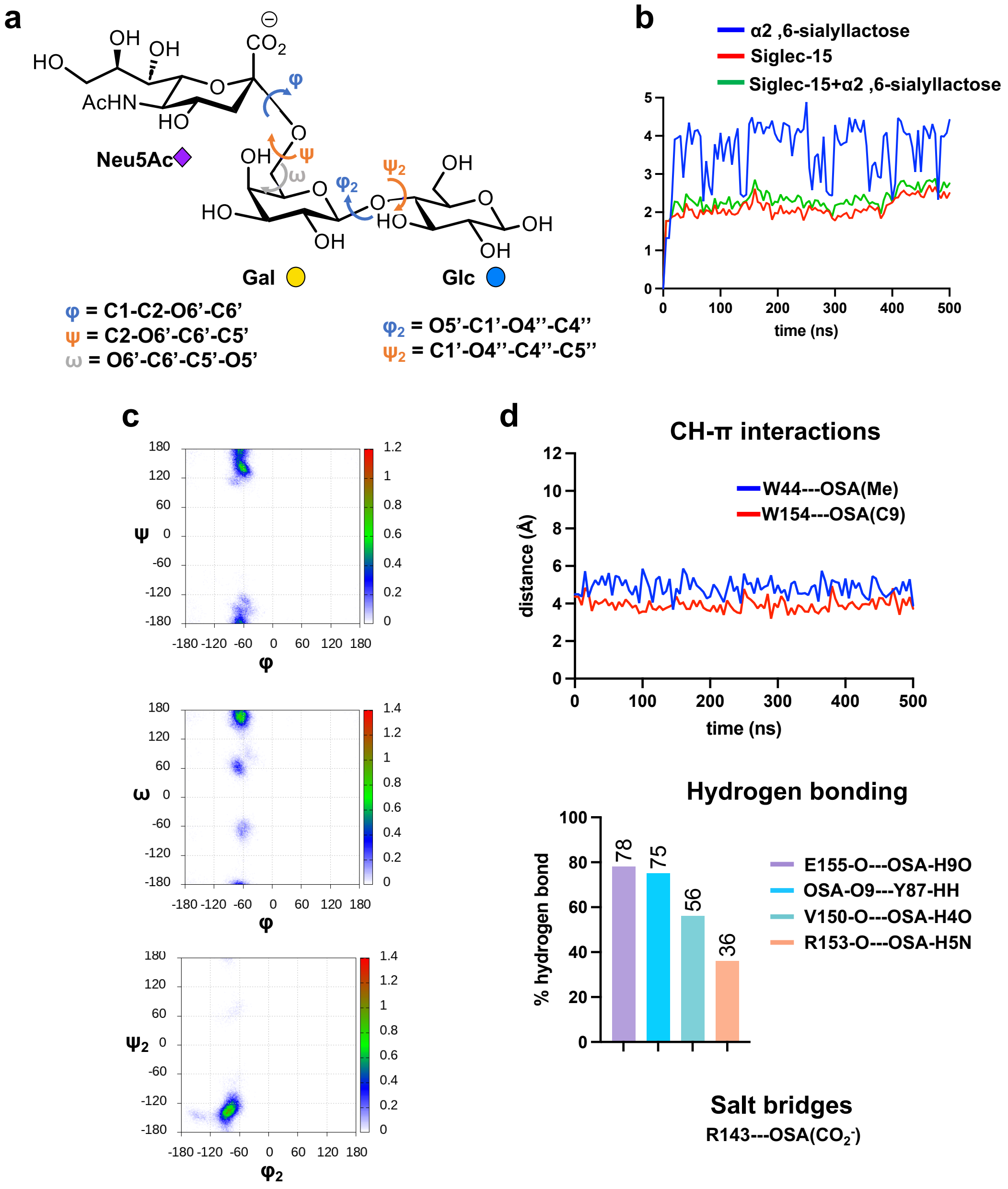

Supplementary Fig. 13. MD simulations of Siglec-15 in complex with 6'SL. a Structure of 6'SL along with the definition of the torsion angles Φ/ψ/ω and $Φ_2/ψ_2$. **b** RMSD (root-mean-square deviation) plots for 6'SL, Siglec-15 and complex Siglec-15/6'SL along 500 ns MD simulations. **c** Φ/ψ/ω distribution around Neu5Acα(2-6)-GalNAc linkage and $Φ_2/ψ_2$ distribution around Galβ(1-4)Glc linkage during 500 ns MD simulations. **d** Molecular interactions (CH-π, hydrogen bonding, salt bridges) along 500 ns MD simulations. The center of the aromatic ring and the center of the sugar were considered in these calculations.

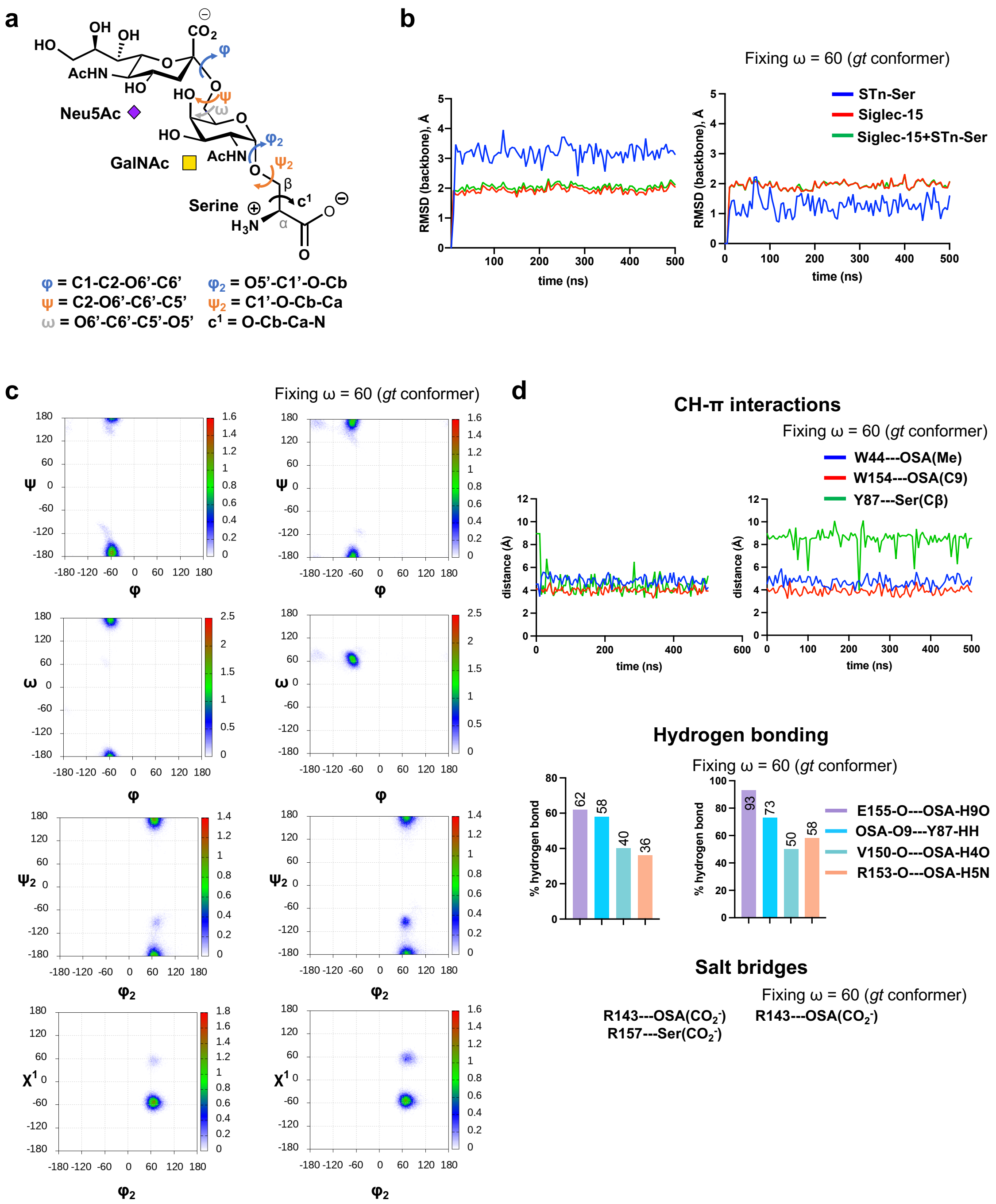

Supplementary Fig. 14. MD simulations of Siglec-15 in complex with STn-Ser. a Structure of STn-Ser along with the definition of the torsion angles Φ/ψ/ω and $Φ_2/ψ_2/χ^1$. **b** RMSD (root-mean-square deviation) plots for STn-Ser, Siglec-15 and complex Siglec-15/STn-Ser along 500 ns MD simulations. **c** Φ/ψ/ω distribution around Neu5Acα(2-6)-GalNAc linkage and $Φ_2/ψ_2/χ^1$ distribution around αGalNAc-*O*-Ser linkage during 500 ns MD simulations. **d** Molecular interactions (CH-π, hydrogen bonding, salt bridges) along 500 ns MD simulations. The center of the aromatic ring and the center of the sugar were considered in these calculations. It is noteworthy that

restraining the value of w results in the loss of the CH-π interaction between Y87 and the Cβ of the serine residue, as well as the salt bridge between R157 and Ser (CO_2^-).

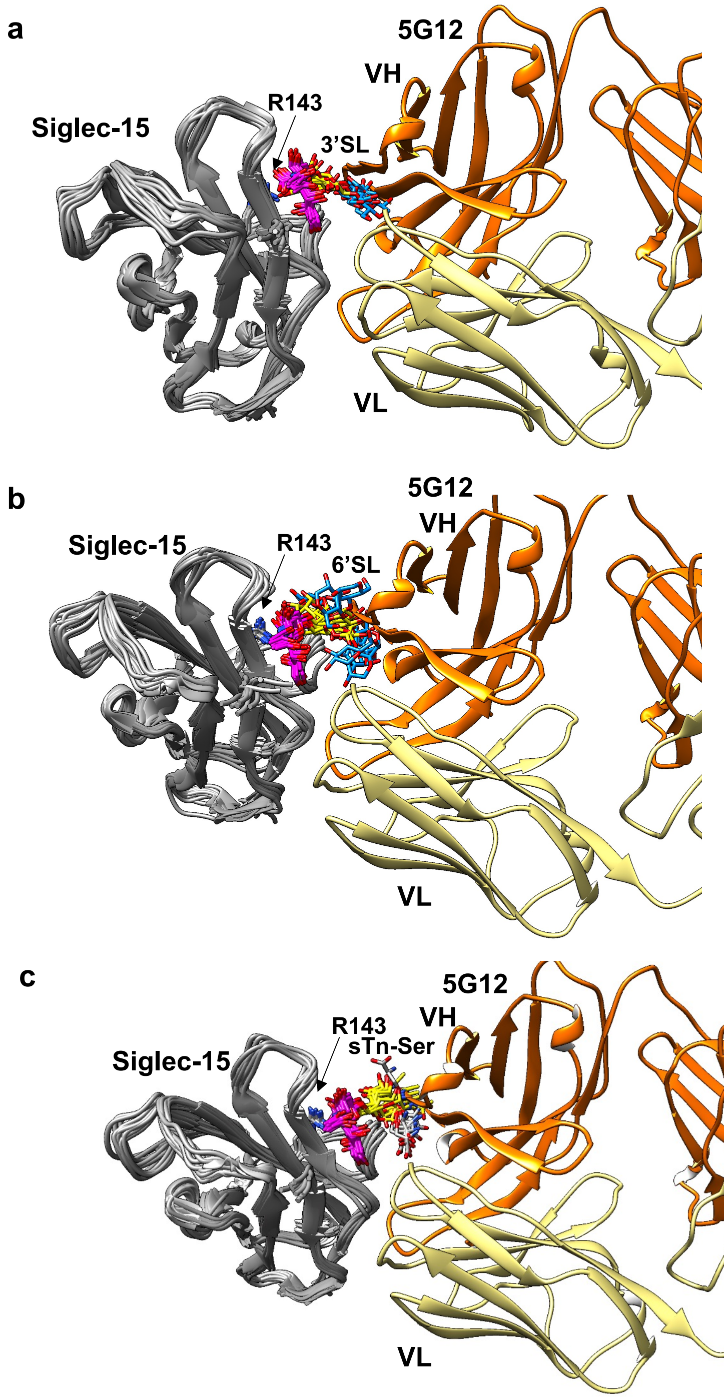

Supplementary Fig. 15. Cartoon representation to illustrate that 5G12 competes to 3'SL, 6'SL and STn-Ser with Siglec-15. Alignment of the structure 10 frames obtained from the 500 ns MD simulations of 3'SL/Siglec-15 (A), 6'SL/Siglec-15 (B) or STn-Ser/Siglec-15 (C) and the crystal structure of Siglec-15 (dark gray) in complex with 5G12 mAb (orange and yellow).

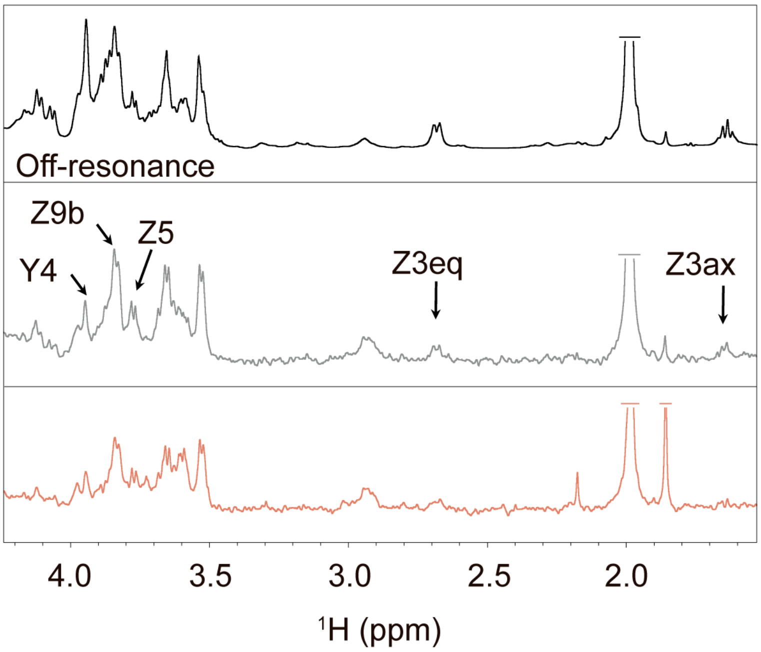

Supplementary Fig. 16. Competition STD-NMR experiments showing the reference off-resonance spectrum (top, black) and spectra of Siglec-15 + sTn-Ser mixture (molar ratio 1/20) (molar ratio 1/20/0.05) (middle, gray) and Siglec-15 + sTn-Ser + CD11b/CD18 mixture (molar ratio 1/20/0.05) (bottom, red).

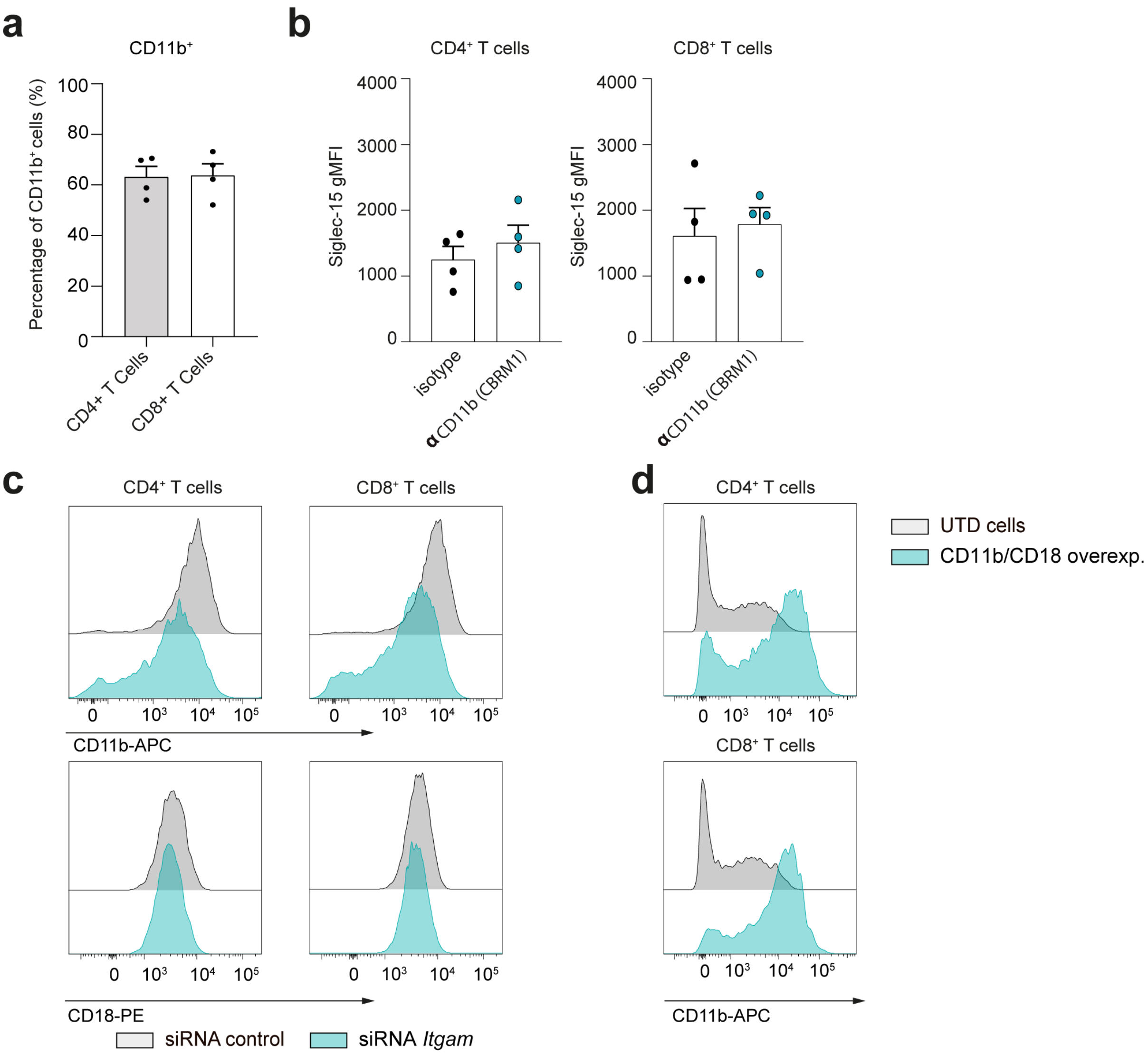

Supplementary Fig.17. Assessing binding of Siglec-15 to CD11b⁺ T cells. a The percentages of expression of CD11b⁺ on CD4⁺ or CD8⁺ T cells after 6 days of activation are shown in the bar graph (n=4 donors). **b** Levels of Siglec-15 binding to CD4⁺ or CD8⁺ human T cells are shown after incubation with isotype control mAb (white bars) or anti-CD11b mAb clone CBRM1 (green bars), (n=4 donors). **c** Cell surface expression levels of CD11b and CD18 in transfected human CD4⁺ and CD8⁺ T cells. **d** Cell surface expression levels of CD11b in transduced T cells. Errors bars denote SEM. ns= non-significant as determined by two-tailed, unpaired Student's t test.

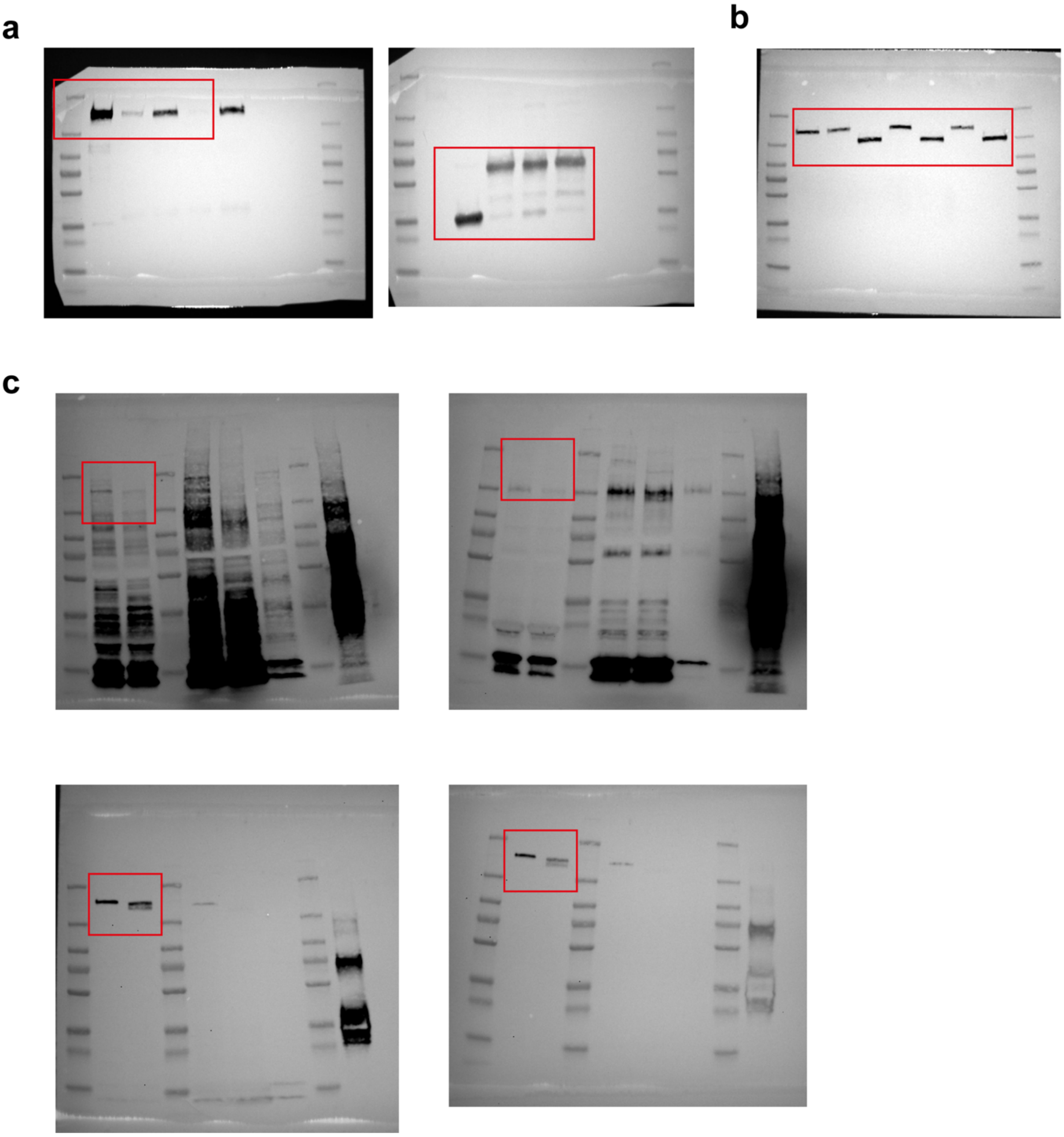

Supplementary Fig. 18. Complete western blot membranes. a Co-IP membranes from figure 5b**. b** Western blot of CD11b on T cell lysates treated with PNGase F, O-glycosidases or Neuraminidase A for 4 hours as indicated in Figure 6A. **c** Lectin blot analysis of purified CD11b-flag from transduced human CD3$^+$ T cells as shown in Figure 6B.

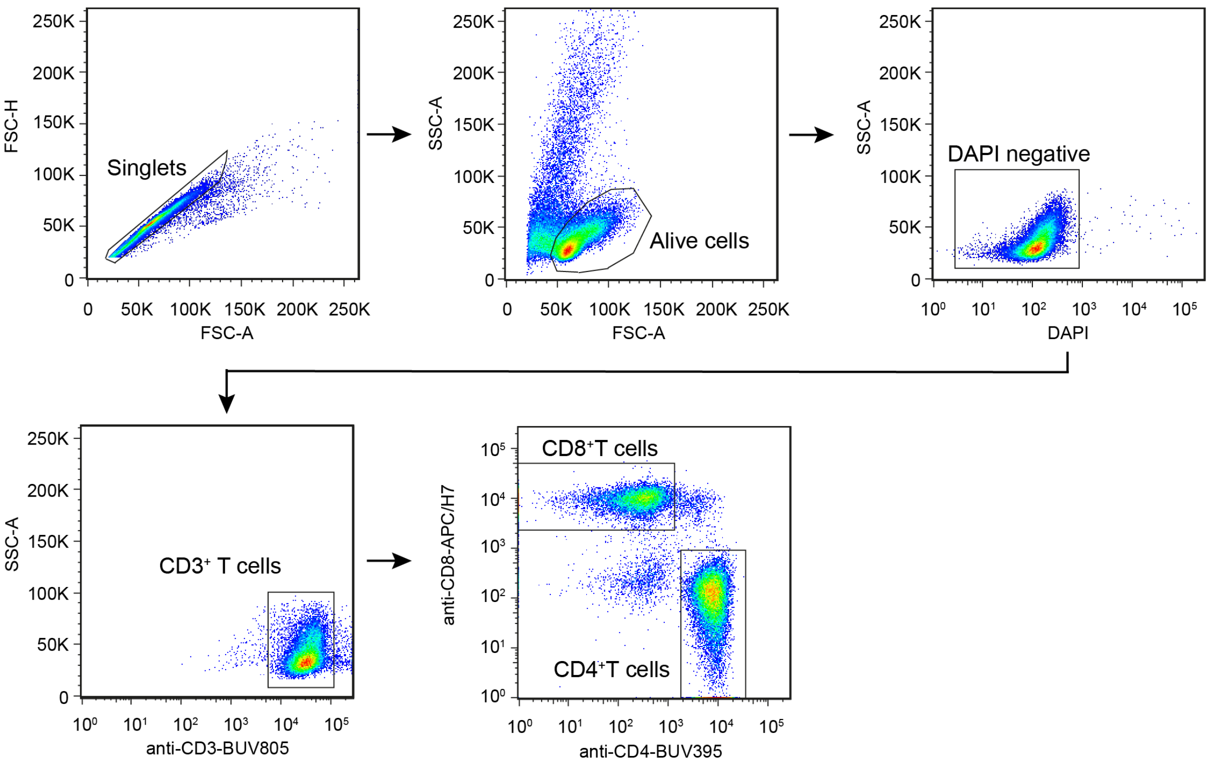

Supplementary Fig. 19. Representative flow cytometry gating.

Supplementary Table 1. Crystallographic data collection and refinement statistics.

	5G12	Siglec-15-5G12
PDB ID	7ZOR	7ZOZ
Data collection Statistics		
Wavelength (Å)	0.97926	0.97925
Resolution range (Å)	62.47-3.93 (4.00-3.93)	106.34-2.10 (2.14- 2.10)
Space group	C121	C121
Unit cell a, b, c (Å)<br>α, β, γ (°)	127.02, 60.68, 131.26<br>90.00, 107.86, 90.00	216.53, 60.53 53.37<br>90.00, 100.82, 90.00
Total reflections	53,013	240,835
Unique reflections	8,690 (429)	37,172 (1,824)
Multiplicity	6.1 (4.4)	6.5 (4.9)
Completeness (%)	99.9 (99.9)	93.8 (91.8)
Mean I/σI	6.4 (3.2)	15.1 (2.3)
Wilson B-factor (Å2)	48.69	33.79
Rmerge	0.465 (0.931)	0.078 (0.633)
Rpim	0.208 (0.552)	0.033 (0.307)
CC1/2	86.5 (51.1)	99.8 (83.5)
Refinement Statistics		
Resolution (Å)	62.47-3.93 (4.00-3.93)	106.34-2.10 (2.14- 2.10)
Rwork	0.229 (0.241)	0.176 (0.246)
Rfree	0.273 (0.298)	0.218 (0.314)
Number of non-hydrogen atoms:<br>macromolecules<br>ligands<br>solvent 	 6,728<br>6,728<br>-<br>-	 4,752<br>4,631<br>35<br>86
RMS (bonds)	0.002	0.004
RMS (angles)	0.482	1.02
Ramachandran statistics:<br>favored (%)<br>allowed (%)<br>outliers (%)	 97.2<br>2.8<br>0.0	 97.7<br>2.3<br>0.0
Rotamer outliers (%)	0.0	0.0
Average B (Å2)<br>macromolecules<br>ligands<br>solvent	 51.2<br>-<br>-	 40.0<br>-<br>47.5
Clash score	6.3	3.4

Supplementary Table 2. Residues involved in the 5G12-Siglec-15 interactions. S= salt bridge; H= hydrogen bond.

Contact type	Siglec-15 residue	BSA (Å^2)
HS	R43	109
	W44	8
	S45	4
	M46	13
	P50	32+38
	E51	3
	G84	1
H	E85	63
	P86	30
	Y87	28+78
	A88	54
HS	R139	64
	F141	5
H	Y154	30
H/HS	R157	68+76
S	H158	85+54
H	G159	10
H	R161	41
	Total	894
	5G12 HC residue	BSA (Å^2)
	S31	4
H	W33	43
	D50	2
	Y52	32
	S55	10
	T58	31
	H60	41
	W111	9
HS	D113	25
	Y114	53
H	G115	67
H	S116	36
	S117	47
	Y118	29
HS	D119	19
	Total	448
	5G12 LC residue	BSA (Å^2)
HS	D1	24
	I2	1
	Q27	35
	N30	12
	Y32	48
	R51	28
	Y94	28
	D95	20
HS	E96	67
	F94	47
H	Y99	24
	Total	334

Supplementary Table 3. List of counter binders for Siglec-15 expressed in human T cells identified by proximity labelling. N=3 biologically independent experiments.

Gene	Protein ID	Protein	Peptides		PSMs		
			Mut	Wt	Mut	Wt	Wt/Mut
IGHA1	P01876	Immunoglobulin heavy constant alpha 1	0	9	0	73	Excl Wt
STATH	P02808	Statherin	3	4	9	70	7,78
IGKC	P01834	Immunoglobulin kappa constant	0	5	0	51	Excl Wt
S100A9	P06702	Protein S100-A9	2	7	20	49	2,45
TF	P02787	Serotransferrin	0	7	0	37	Excl Wt
PIGR	P01833	Polymeric immunoglobulin receptor	0	7	0	33	Excl Wt
S100A8	P05109	Protein S100-A8	2	4	9	23	2,56
IGHG1	P01857	Immunoglobulin heavy constant gamma 1	0	3	0	18	Excl Wt
IGHV3-13	P01766	Immunoglobulin heavy variable 3-13	0	1	0	17	Excl Wt
IGLC2	P0DOY2	Immunoglobulin lambda constant 2	0	3	0	14	Excl Wt
LGALS7	P47929	Galectin-7	0	3	0	13	Excl Wt
JCHAIN	P01591	Immunoglobulin J chain	0	2	0	12	Excl Wt
IGKV2D-29	A0A075B6S2	Immunoglobulin kappa variable 2D-29	0	2	0	10	Excl Wt
HLA-A	P04439	HLA class I histocompatibility antigen, A alpha chain	1	2	1	10	10,00
MUC5B	Q9HC84	Mucin-5B	0	2	0	8	Excl Wt
ATP1A3	P13637	Sodium/potassium-transporting ATPase subunit alpha-3	0	1	0	6	Excl Wt
IGKV3D-11	A0A0A0MRZ8	Immunoglobulin kappa variable 3D-11	0	1	0	5	Excl Wt
IGHV3-66	A0A0C4DH42	Immunoglobulin heavy variable 3-66	0	1	0	5	Excl Wt
ADGRA3	Q8IWK6	Adhesion G protein-coupled receptor A3	1	1	1	5	5,00
CP	P00450	Ceruloplasmin	0	2	0	5	Excl Wt
CD44	P16070	CD44 antigen	0	2	0	5	Excl Wt
IGHM	P01871	Immunoglobulin heavy constant mu	0	2	0	4	Excl Wt
ITGB2	P05107	Integrin beta-2	0	1	0	4	Excl Wt
IGHA2	P01877	Immunoglobulin heavy constant alpha 2	0	1	0	4	Excl Wt
SLC4A2	P04920	Anion exchange protein 2	0	1	0	4	Excl Wt
PAICS	P22234	Multifunctional protein ADE2	0	2	0	4	Excl Wt
CALR	P27797	Calreticulin	0	3	0	4	Excl Wt
NME2P1	O60361	Putative nucleoside diphosphate kinase	0	2	0	3	Excl Wt
IGKV3-15	P01624	Immunoglobulin kappa variable 3-15	0	1	0	3	Excl Wt
IGLV1-47	P01700	Immunoglobulin lambda variable 1-47	0	1	0	3	Excl Wt
ATP1A4	Q13733	Sodium/potassium-transporting ATPase subunit alpha-4	0	1	0	2	Excl Wt
KIDINS220	Q9ULH0	Kinase D-interacting substrate of 220 kDa	0	1	0	2	Excl Wt
IGLC7	A0M8Q6	Immunoglobulin lambda constant 7	0	1	0	2	Excl Wt
HLA-E	P13747	HLA class I histocompatibility antigen, alpha chain E	0	1	0	2	Excl Wt
ITGAM	P11215	Integrin alpha-M	0	1	0	2	Excl Wt
TMPRSS13	Q9BYE2	Transmembrane protease serine 13	0	1	0	2	Excl Wt
IGKV3D-20	A0A0C4DH25	Immunoglobulin kappa variable 3D-20	0	1	0	1	Excl Wt
MS4A10	Q96PG2	Membrane-spanning 4-domains subfamily A member 10	0	1	0	1	Excl Wt

PDZD2	O15018	PDZ domain-containing protein 2	0	1	0	1	Excl Wt
ORM1	P02763	Alpha-1-acid glycoprotein 1	0	1	0	1	Excl Wt
AHSG	P02765	Alpha-2-HS-glycoprotein	0	1	0	1	Excl Wt
CCDC168	Q8NDH2	Coiled-coil domain-containing protein 168	0	1	0	1	Excl Wt
IGLV3-9	A0A075B6K5	Immunoglobulin lambda variable 3-9	0	1	0	1	Excl Wt
TRBV4-1	A0A577	T cell receptor beta variable 4-1	0	1	0	1	Excl Wt
IGKV3-20	P01619	Immunoglobulin kappa variable 3-20	0	1	0	1	Excl Wt
MSN	P26038	Moesin	0	1	0	1	Excl Wt
VLDLR	P98155	Very low-density lipoprotein receptor	0	1	0	1	Excl Wt
SHROOM3	Q8TF72	Protein Shroom3	0	1	0	1	Excl Wt
RYR2	Q92736	Ryanodine receptor 2	0	1	0	1	Excl Wt
PHB2	Q99623	Prohibitin-2	0	1	0	1	Excl Wt
SYTL2	Q9HCH5	Synaptotagmin-like protein 2	0	1	0	1	Excl Wt

Table shows the gene names, uniprot protein IDs and protein names. The total number of distinct peptide sequences, and the number of peptide spectrum matches (PSMs) identified in each protein is shown. Siglec-15 R143A mutant was used as a control. Excl Wt= exclusively identified after proximity labelling performed with WT Siglec-15.

SUPPLEMENTARY REFERENCES

1 Schrödinger, L. The PyMol MolecularGraphics System, Versión 1.8. *Thomas Hold* (2015).

2 Ohrui, H., Nishida, Y., Itoh, H. & Meguro, H. Preferred conformation about the C5-C6 bond of N-acetylneuraminyl(2-6)-D-galacto- and -D-glucopyranosides in solution. *The Journal of Organic Chemistry* **56**, 1726-1731 (1991).
