# Supplementary material for: Structural insights into Siglec-15 reveal glycosylation dependency for its interaction with T cells through integrin CD11b
Source: Nat Commun. 2023 Jun 13;14:3496. doi: 10.1038/s41467-023-39119-8 (PMC10264397; doi:10.1038/s41467-023-39119-8)
Supplement: Supplementary file 3 — Reporting Summary [file 41467_2023_39119_MOESM3_ESM.pdf]

nature portfolio

Corresponding author(s): Jesús Jiménez-Barbero, Asis Palazon, June Ereño-Orbea

Last updated by author(s): May 10, 2023

Reporting Summary

Nature Portfolio wishes to improve the reproducibility of the work that we publish. This form provides structure for consistency and transparency in reporting. For further information on Nature Portfolio policies, see our Editorial Policies and the Editorial Policy Checklist.

Statistics

For all statistical analyses, confirm that the following items are present in the figure legend, table legend, main text, or Methods section.

n/a	Confirmed	
☐	☒	The exact sample size (*n*) for each experimental group/condition, given as a discrete number and unit of measurement
☐	☒	A statement on whether measurements were taken from distinct samples or whether the same sample was measured repeatedly
☐	☒	The statistical test(s) used AND whether they are one- or two-sided<br>*Only common tests should be described solely by name; describe more complex techniques in the Methods section.*
☐	☒	A description of all covariates tested
☐	☒	A description of any assumptions or corrections, such as tests of normality and adjustment for multiple comparisons
☐	☒	A full description of the statistical parameters including central tendency (e.g. means) or other basic estimates (e.g. regression coefficient) AND variation (e.g. standard deviation) or associated estimates of uncertainty (e.g. confidence intervals)
☐	☒	For null hypothesis testing, the test statistic (e.g. *F*, *t*, *r*) with confidence intervals, effect sizes, degrees of freedom and *P* value noted<br>*Give P values as exact values whenever suitable.*
☒	☐	For Bayesian analysis, information on the choice of priors and Markov chain Monte Carlo settings
☐	☒	For hierarchical and complex designs, identification of the appropriate level for tests and full reporting of outcomes
☒	☐	Estimates of effect sizes (e.g. Cohen's *d*, Pearson's *r*), indicating how they were calculated

Our web collection on statistics for biologists contains articles on many of the points above.

Software and code

Policy information about availability of computer code

Data collection	FACSymphony, FACSAria Fusion, NIVO, iBright, Octet BLI Discovery 12.2.2.20, Octet Analysis Studio 12.2.2.26, AKTA start, AKTA pure, Bruker Avance IIII 600 MHz spectometrer, Bruker Avance III 800 MHz spectometer, TopSpin 3.6.5, UNICORN 7, Amber 20 package, HyStar Version 5.1.8.1
Data analysis	FlowJo V10.7.1. (BD biosciences), XDS, PISA, Phenix, Coot, Pymol 1.7x, AutoDock Vina 1.5.7, AMBER package (v20), uGLYCAM06j-168, Octet software (Sartorius), Byonic software (v2.16.11, Protein Metrics), Prism v8 (Graphpad),WinCoot 0.8.9.2, ff14SB, GLYCAM06j, Matrix Science v2.2.07

For manuscripts utilizing custom algorithms or software that are central to the research but not yet described in published literature, software must be made available to editors and reviewers. We strongly encourage code deposition in a community repository (e.g. GitHub). See the Nature Portfolio guidelines for submitting code & software for further information.

Data

Policy information about availability of data

All manuscripts must include a data availability statement. This statement should provide the following information, where applicable:

- Accession codes, unique identifiers, or web links for publicly available datasets
- A description of any restrictions on data availability
- For clinical datasets or third party data, please ensure that the statement adheres to our policy

Data availability

All data generated or analysed during this study (and its supplementary information files) are included in this published article. Any remaining information can be obtained from the corresponding author upon reasonable request.
The crystalographic data of 5G12 Fab and Siglec15–5G12 Fab complex generated in this study have been deposited in the Protein Data Bank database under accession codes 7ZOZ and 7ZOR [].
The mass spectrometry proteomics data have been deposited to the ProteomeXchange Consortium via the PRIDE partner repository with the dataset identifier PXD042009".
Molecular dynamics simulations data, along with the top 10 poses from AutoDock Vina for Siglec15+STn-OMe, have been deposited in the "open science framework" repository and can be accessed at the following link: https://osf.io/ykgf5/?view_only=21f8c01e396b456dadba577a842f49d9.

Human research participants

Policy information about studies involving human research participants and Sex and Gender in Research.

Reporting on sex and gender	*Use the terms sex (biological attribute) and gender (shaped by social and cultural circumstances) carefully in order to avoid confusing both terms. Indicate if findings apply to only one sex or gender; describe whether sex and gender were considered in study design whether sex and/or gender was determined based on self-reporting or assigned and methods used. Provide in the source data disaggregated sex and gender data where this information has been collected, and consent has been obtained for sharing of individual-level data; provide overall numbers in this Reporting Summary. Please state if this information has not been collected. Report sex- and gender-based analyses where performed, justify reasons for lack of sex- and gender-based analysis.*
Population characteristics	*Describe the covariate-relevant population characteristics of the human research participants (e.g. age, genotypic information, past and current diagnosis and treatment categories). If you filled out the behavioural & social sciences study design questions and have nothing to add here, write "See above."*
Recruitment	*Describe how participants were recruited. Outline any potential self-selection bias or other biases that may be present and how these are likely to impact results.*
Ethics oversight	*Identify the organization(s) that approved the study protocol.*

Note that full information on the approval of the study protocol must also be provided in the manuscript.

Field-specific reporting

Please select the one below that is the best fit for your research. If you are not sure, read the appropriate sections before making your selection.

[x] Life sciences [ ] Behavioural & social sciences [ ] Ecological, evolutionary & environmental sciences

For a reference copy of the document with all sections, see nature.com/documents/nr-reporting-summary-flat.pdf

Life sciences study design

All studies must disclose on these points even when the disclosure is negative.

Sample size	No statistical power test were used to determine sample size needed. At least three biological replicates were analyzed and different donors were used to account for variation. The exact n numbers used in each experiment are indicated in the figure legends.
Data exclusions	No data was excluded from the study
Replication	In vitro experiments were typically run with triplicate biological replicates and most of the experiments were reproduced at least twice.
Randomization	For in vitro studies, samples were allocated to identical cell culture systems and there is no reason to believe that spatial organization influenced experimental outcomes.
Blinding	Studies on human PBMCs were performed without blinding. Data were analyzed by software with objective outcomes and quantification was performed in an uniform manner for all samples tested. Therefore, blinding was not relevant for this study.

Reporting for specific materials, systems and methods

We require information from authors about some types of materials, experimental systems and methods used in many studies. Here, indicate whether each material, system or method listed is relevant to your study. If you are not sure if a list item applies to your research, read the appropriate section before selecting a response.

Materials & experimental systems

n/a	Involved in the study	
	x	Antibodies
	x	Eukaryotic cell lines
x		Palaeontology and archaeology
x		Animals and other organisms
x		Clinical data
x		Dual use research of concern

Methods

n/a	Involved in the study	
x		ChIP-seq
	x	Flow cytometry
x		MRI-based neuroimaging

Antibodies

Antibodies used

Fluorochrome-labeled antibodies:

-anti-CD3 BUV805 (612894, BD Biosciences; 1:100), https://www.bdbiosciences.com/en-ca/products/reagents/flow-cytometry-reagents/research-reagents/single-color-antibodies-ruo/buv805-mouse-anti-human-cd3.612894
-anti-CD4 BUV395 (563550, BD Biosciences; 1:200) https://www.bdbiosciences.com/en-us/products/reagents/flow-cytometry-reagents/research-reagents/single-color-antibodies-ruo/buv395-mouse-anti-human-cd4.563550
-anti-CD8 APC/H7 (566855, BD Biosciences; 1:200) https://www.bdbiosciences.com/en-us/products/reagents/flow-cytometry-reagents/research-reagents/single-color-antibodies-ruo/apc-h7-mouse-anti-human-cd8.566855
-anti-CD8 BUV805 (612889, BD Biosciences) https://www.bdbiosciences.com/en-us/products/reagents/flow-cytometry-reagents/research-reagents/single-color-antibodies-ruo/buv805-mouse-anti-human-cd8.612889
-streptavidin PE (12-4317-87, Thermo Fisher; 1:200), https://www.thermofisher.com/order/catalog/product/es/es/12-4317-87
-anti-STn primary antibody (ab115957, Abcam; 1:100), https://www.abcam.com/products/primary-antibodies/sialyl-tn-antibody-stn-219-ab115957.html
-anti-mouse IgG-FITC secondary antibody (406001, Biolegend; 1:200),https://www.biolegend.com/en-us/products/fitc-goat-anti-mouse-igg-minimal-x-reactivity-1391?GroupID=BLG2049
-anti-human IgG Fc PE (12-4998-82, Thermo Fisher), https://www.thermofisher.com/antibody/product/Goat-anti-Human-IgG-Fc-Secondary-Antibody-Polyclonal/12-4998-82
-anti-CD11b biotin antibody (clone M1/70, 557394, BD Biosciences) https://www.bdbiosciences.com/en-us/products/reagents/flow-cytometry-reagents/research-reagents/single-color-antibodies-ruo/biotin-rat-anti-cd11b.553309
-anti-CD11b biotin antibody (clone CBRM1/5, 14-0113-81, Thermo Fisher)https://www.thermofisher.com/antibody/product/CD11b-activation-epitope-Antibody-clone-CBRM1-5-Monoclonal/14-0113-81

Secondary HRP-Conjugated antibodies:
-anti-human Fc HRP (A01854200, Genscript) https://www.genscript.com/antibody/A01854-Mouse_Anti_Human_IgG_Fc_Antibody_HRP_mAb.html
-anti-rabbit IgG HRP (7074S, cell signaling) https://www.cellsignal.com/products/secondary-antibodies/anti-rabbit-igg-hrp-linked-antibody/7074

Non-labeled antibodies:

-anti-CD11b (ab133357, Abcam) https://www.abcam.com/products/primary-antibodies/cd11b-antibody-epr1344-ab133357.html
-Isotype control rIgG2a (14-4321-82, Thermo Fisher) https://www.thermofisher.com/antibody/product/Rat-IgG2a-kappa-clone-eBR2a-Isotype-Control/14-4321-82
-Isotype control mIgG1, (14-4714-82, Thermo Fisher) https://www.thermofisher.com/antibody/product/Mouse-IgG1-kappa-clone-P3-6-2-8-1-Isotype-Control/14-4714-82

Validation

All antibodies were validated for the use of flow cytometry. Data are available on the manufacturers' websites. For experiments shown in this study, single-stain controls and an unstained control were used to assess staining of experimental samples.

Eukaryotic cell lines

Policy information about cell lines and Sex and Gender in Research

Cell line source(s)	The lentiviral packaging cell line Lenti-X™ 293T was purchased from Takarabio. HEK293F was purchased from Thermo Fisher. HEK293S, Jurkat cells and K562 cells were purchased from ATCC.
Authentication	All cell lines have been authenticated by STR profiling by the corresponding company.
Mycoplasma contamination	All cell lines tested negative for mycoplasma contamination.
Commonly misidentified lines (See ICLAC register)	None of the cell lines used in this study are listed in the ICLAC database of Cross-Contaminated or Misidentified cell lines

Flow Cytometry

Plots

Confirm that:

- [x] The axis labels state the marker and fluorochrome used (e.g. CD4-FITC).
- [x] The axis scales are clearly visible. Include numbers along axes only for bottom left plot of group (a 'group' is an analysis of identical markers).
- [x] All plots are contour plots with outliers or pseudocolor plots.
- [x] A numerical value for number of cells or percentage (with statistics) is provided.

Methodology

Sample preparation	For analysis of CD11b expression on T cells, activated T cells were collected at day 8, washed in Flow Cytometry Staining Buffer (00-4222-26, Thermo Fisher) and incubated with anti-CD11b biotin antibody (553309, BD Biosciences; 1:100) for 30 min at 4 ºC. Cells where then washed and incubated with anti-CD3 BUV805 (612894, BD Biosciences; 1:100), anti-CD4 BUV395 (563550, BD Biosciences; 1:200) anti-CD8 APC/H7 (566855, BD Biosciences; 1:200) and streptavidin PE (12-4317-87, Thermo Fisher; 1:200) for 30 min at 4 ºC in the dark. After washing, cells were resuspended in 200 µL staining buffer containing DAPI (1:10000) (D1306, Invitrogen). For binding of recombinant proteins, cells were incubated with recombinant Siglec-15-Fc, Siglec-15-Fc R143A mutant or human IgG1Fc control (110-HG-100, R&D) at 4 µg/mL for 30 min at 4 ºC. Cells were then washed and incubated with anti-human IgG Fc PE (12-4998-82, Thermo Fisher), anti-CD4 BUV395 (563550, BD Biosciences) and anti-CD8 BUV805 (612889, BD Biosciences) and incubated for 30 min at 4 ºC in the dark. Cells were washed and resuspended in 200 µL 2 % BSA in PBS with DAPI (1:10000) before acquisition on a FACSymphony.
Instrument	FACSymphony cytometer or FACSAria Fusion
Software	FlowJo software
Cell population abundance	When CD11b+ T cells were sorted the purity of the cell population was above 90%.
Gating strategy	Singlets FSC/SSC>DAPI negative cells>CD3+ T cells>CD4 or CD8+ T cells>CD11b+ T cells or binding of recombinant proteins

- [x] Tick this box to confirm that a figure exemplifying the gating strategy is provided in the Supplementary Information.
